# Muscle-Derived BMP4 Regulates Morphology and Function of Endplates on Extrafusal and Intrafusal Muscle Fibers in Adult Mice

**DOI:** 10.1523/JNEUROSCI.0707-25.2025

**Published:** 2025-10-09

**Authors:** Julia M. Harrison, Borbala Podor, Asal Yans, Victor F. Rafuse

**Affiliations:** ^1^Department of Medical Neuroscience, Dalhousie University, Halifax, Nova Scotia B3H 1X5, Canada; ^2^Brain Repair Centre, Life Sciences Research Institute, Halifax, Nova Scotia B3H 4R2, Canada

**Keywords:** behavior, muscle, neuromuscular junction, neurotransmission, plasticity, proprioception

## Abstract

Understanding factors contributing to neuromuscular junction (NMJ) stability postdevelopment will shed light on how this stability is lost during aging and in neuromuscular diseases. Previous work in *Drosophila* suggests that morphogens within the bone morphogenic protein (BMP) family are potential candidates because the BMP homolog, *gbb*, along with its receptor, *wit*, have key roles in NMJ structure, stability, and function. Whether BMPs have similar roles at vertebrate NMJs is currently unknown. To examine this question, we generated doxycycline-inducible, muscle-specific BMP4 null mice, referred to here as HSA^Cre^BMP4^fl/fl^ mice. Motor behavior tasks were examined pre- and postinduction while electrophysiological and morphological characteristics were examined 4 months later in mice of both sexes. Soleus muscles from HSA^Cre^BMP4^fl/fl^ mice had significantly reduced contractile force compared with wild-type (WT) littermates. Cross-sectional areas of type I, but not type IIa, muscle fibers were reduced. NMJs were also larger in HSA^Cre^BMP4^fl/fl^ muscles compared with controls due to a significant increase in acetylcholine receptor fragment number and distribution. HSA^Cre^BMP4^fl/fl^ NMJs displayed reduced amplitude and frequency of miniature endplate potentials (mEPPs), evoked EPP amplitude, and quantal content and had increased failure rates when stimulating at high frequencies. Behaviorally, HSA^Cre^BMP4^fl/fl^ mice performed increasingly worse over time on the rotarod after doxycycline administration compared with their WT littermates. Finally, muscle spindle structure and proprioceptive function were significantly compromised in HSA^Cre^BMP4^fl/fl^ mice. These results indicate that muscle-derived BMP4 regulates morphological and electrophysiological attributes of the NMJ in adult mice as well as the structure and function of muscle spindles.

## Significance Statement

Understanding the cellular mechanisms underlying neuromuscular junction (NMJ) stability is critically important in understanding why it is compromised during aging and in motoneuron diseases. Studies in *Drosophila* larvae have shown that *gbb* and *wit*, a ligand and receptor in the BMP signaling pathway, are critical for the stability and function of the NMJ. This paper uses a novel doxycycline-inducible, muscle-specific BMP4 knockdown approach to eliminate muscular BMP4 expression in adult mice. When BMP4 was excised in the adult, we found that muscle strength and neurotransmission were attenuated, endplates were fragmented, and mice had locomotor deficits. Furthermore, muscle spindle innervation and proprioceptive function were impaired. Therefore, as in *Drosophila* larvae, BMP4 is required for normal function and morphology of adult vertebrate NMJs.

## Introduction

The formation and maturation of neuromuscular junctions (NMJs) is regulated by multiple anterograde and retrograde signals between the innervating motoneurons, terminal Schwann cells (TSCs), and postsynaptic muscle fibers (Sanes and Lichtman, 2001; Wu et al., 2010). Once established, NMJs typically persist throughout life. This persistence is due, in part, to homeostatic plasticity between the motoneuron and muscle fibers, enabling the NMJ to remodel in response to destabilizing perturbations such as aging, injury, and disease (Sanes and Lichtman, 2001). Remodeling can include changes in ion channel expression, postsynaptic acetylcholine receptor (AChR) numbers and distribution, as well as modulating neurotransmitter release (Davis and Müller, 2015). When destabilizations are extreme, as occurs in amyotrophic lateral sclerosis (ALS), NMJs become unstable, leading to the cessation of neurotransmission, motor axon withdrawal, muscle weakness, and paralysis (Arbour et al., 2017).

Several pre- and postsynaptic components involved in NMJ development have been shown to contribute to its stability, including agrin, muscle-specific tyrosine kinase (MuSK), low-density lipoprotein receptor-related protein 4 (LRP4), extracellular matrix proteins, adhesion molecules, and growth factors (Bloch-Gallego, 2015; Takamori, 2017). Additionally, morphogens, such as the bone morphogenic protein (BMP) family of molecules, have been shown to modulate neurotransmitter release and endplate size. *Drosophila* larvae with mutations in glass bottom boat (*gbb*) and wishful thinking (*wit*), the *Drosophila* homologs to BMP and its receptor, BMPRII, respectively, have pre- and postsynaptic defects at the NMJ (Aberle et al., 2002; Marqués et al., 2002; McCabe et al., 2003, 2004; Goold and Davis, 2007). Genetic deletion of *wit* resulted in severe motor defects and smaller NMJs with aberrant synaptic bouton ultrastructure. Synaptic deficits included reductions in the size and frequency of spontaneous miniature excitatory junctional potentials (mEJPs), smaller evoked EJPs, and decreased quantal content (Aberle et al., 2002; Marqués et al., 2002). *Wit* mutations also caused motoneuron retraction from the NMJ resulting in severe motor deficits including poor wing and leg movement and an inability to exit the pupal case, causing death (Marqués et al., 2002; Lee et al., 2016). Interestingly, restoration of *wit* in motoneurons, but not muscles, rescued all phenotypes caused by ubiquitous *wit* loss (Aberle et al., 2002; Marqués et al., 2002). Genetic deletion of *gbb* in *Drosophila* larvae also resulted in small NMJs, decreased frequency of mEJPs, smaller EJPs, and reduced quantal content. These phenotypes were eliminated when *gbb* was re-expressed in muscles, but not motoneurons, in the *gbb* mutants (McCabe et al., 2003). Taken together, retrograde release of *gbb* from the muscle to activate signaling via *wit* is vital in ensuring the normal health and function of the *Drosophila* NMJ (Osses and Henríquez, 2015).

In contrast, the role of BMP4 signaling at the NMJ in higher vertebrates is poorly understood. In adult mice, the BMP4 receptor, BMPRII, is expressed in adult motoneurons, while BMP4 is expressed in muscle fibers and in Schwann cells (SCs; Son and Thompson, 1995; Chou et al., 2013). Transcripts for many BMP proteins, including BMP4, its receptors, and downstream effectors, have also been identified from single NMJ myonuclei sequencing (Ham et al., 2025). Interestingly, BMP4 is not produced in motoneurons but rather is transported bidirectionally along motor axons (Chou et al., 2013). Stimulation of BMP signaling within muscle fibers downstream of BMPRII has also been shown to be essential for maintaining adult muscle fiber mass (Sartori et al., 2013; Winbanks et al., 2013). Whether BMP4 expression in adult vertebrate muscle regulates neurotransmission and morphology of the NMJ is currently unknown.

To investigate the role of muscular BMP4 at the vertebrate NMJ, we generated inducible, muscle-specific, BMP4 null mice (HSA^Cre^BMP4^fl/fl^) to genetically inhibit its expression starting at approximately postnatal day (P) 90. We found that soleus muscles in HSA^Cre^BMP4^fl/fl^ mice have lower contractile forces, increased endplate fragmentation, reduced neurotransmission, and gamma motoneuron abnormalities compared with wild-type (WT). These mice also performed poorly on motor tasks requiring proprioception. Together, these results indicate that muscular BMP4 is required for normal neuromuscular function in adult mice.

## Materials and Methods

### Mice

Skeletal muscle-specific, doxycycline-inducible, BMP4 knock-out mice (referred to here as HSA^Cre^BMP4^fl/fl^ mice) were generated by crossing B6;C3-Tg(ACTA1-rtTA,tetO-cre)102Monk/J mice (Rao and Monks, 2009; The Jackson Laboratory) with B6;129S4-Bmp4tm1Jfm/J mice (Liu et al., 2004; The Jackson Laboratory). Skeletal muscle-specific, doxycycline-inducible tdTomato mice were generated by crossing B6;C3-Tg(ACTA1-rtTA,tetO-cre)102Monk/J mice with B6.Cg-Gt(ROSA)26Sortm9(CAG-tdTomato)Hze/J (Madisen et al., 2010; The Jackson Laboratory) to generate HSA^Cre^tdTomato^fl/fl^ mice (referred to here as HSA^Cre^ROSATomato^fl/fl^ mice). Cre recombinase activity, under the control of tetO (tetracycline-responsive regulatory element), was induced in both strains following administration of doxycycline in food pellets (Bio-Serv) at ∼P90. Given that human skeletal α-actin (HSA) is selectively expressed in extrafusal and intrafusal skeletal muscles fibers (Leu et al., 2003; Rao and Monks, 2009), induction of Cre recombinase activity caused selective loss of BMP4 expression, and production of tdTomato, in extra- and intrafusal skeletal muscle fibers of >P90 HSA^Cre^BMP4^fl/fl^ and HSA^Cre^ROSATomato^fl/fl^ mice, respectively. HSA Cre-negative, age-matched littermates were used as controls and are referred to as WT throughout. Male and female mice were included in all datasets considering no sex differences were observed following post hoc analysis. All mice appeared healthy and had no observed health abnormalities other than those reported, below. All experiments were conducted in accordance with the guidelines of the Canadian Council on Animal Care and the policies of Dalhousie University.

### Ex vivo isometric force recordings

Mice were anesthetized with a mixture of isoflurane (2–3%) and oxygen. Under aseptic conditions, the soleus or extensor digitorum longus (EDL) muscle, and corresponding nerve supply, were quickly removed and transferred to a Sylgard-coated (Dow Corning) recording chamber that was continuously perfused with carbogenated (95% O_2_ and 5% CO_2_) Tyrode’s solution containing the following (in mM): 125 NaCl, 24 NaHCO_3_, 5.37 KCl, 1 MgCl_2_, 1.8 CaCl_2_, and 27.75 dextrose. A knot was tied around the proximal tendon and secured onto the Sylgard with an insect pin. A suture (6-0) was tied around the distal tendon and attached to a force transducer (FT 03; Grass Technologies). For the EDL, the knot around the tendon was secured with super glue (LOCTITE, Henkel Adhesives) to prevent slippage from the stronger muscle contraction.

A fine-tipped glass stimulating suction electrode was used to deliver electrical current (0.05 ms duration) to the innervating nerve via an S88 stimulator (Grass Technologies) that was isolated from the ground using a stimulus isolation unit (PSIU6; Grass Technologies). Muscle length was adjusted to maximal isometric contraction. Twitch forces were recorded after a single stimulus while tetanic forces were recorded during a 1 s duration, 50 Hz stimulus train for the soleus, and at 100 Hz for the EDL (the EDL required a higher frequency stimulation for a fused tetanus). To characterize whole muscle fatigability, muscles were stimulated at 40 Hz for 350 ms, every second, for 2 min, as previously described (Burke et al., 1973). The fatigue index was determined as the ratio of the force during the last contraction in the series to that of the first. All forces were recorded using a Digidata 1322A analog-to-digital board and AxoScope version 10.2 software (Molecular Devices).

### Intracellular NMJ physiology

Mice were anesthetized with an isoflurane/oxygen mixture (2–3%) to remove the soleus or EDL muscle and supplying nerve. The muscles were preincubated for 90 min in well-oxygenated Tyrode’s solution containing 5 µM conotoxin (CTX) GIIIB (Alomone Labs) to block nerve-evoked muscle contractions (Bewick et al., 2004). The muscles were then pinned in a Sylgard-coated recording chamber perfused with carbogenated normal Tyrode’s solution (see above) for intracellular recordings.

Intracellular recordings of NMJs were performed using sharp recording electrodes (15–30 MΩ) filled with 3 M KCl. Spontaneous miniature endplate potentials (mEPPs) and nerve-evoked transmitter release (endplate potentials; EPPs) were recorded with a MultiClamp 700B amplifier and digitized with MultiClamp Digidata 1140A (Molecular Devices). Nerve-evoked transmitter release was induced by stimulating the soleus or EDL nerve supply with a fine-tipped plastic suction electrode using a Grass S88 stimulator that was isolated from ground using a SIU5 stimulus isolation unit (current range, 1–15 mA). Stimulation pulse width was set at 0.2 ms. Data were acquired with Clampex 10.2 software (Molecular Devices) at a sampling rate of 10 kHz and analyzed using MiniAnalysis software (Synaptosoft). Quantal content (*m*) was determined by the direct method (*m* = mean EPP amplitude/mean mEPP amplitude) from EPPs collected at a 1 Hz stimulation rate (for 10 s) and mEPPs recorded over 30 s (Del Castillo and Katz, 1954). No changes in EPP amplitude or mEPP frequency were observed over recording time. Failures were identified as a stimulus without a subsequent change in resting membrane potential. Paired pulse facilitation (PPR) was determined by stimulating the nerve with pulse intervals of 10 ms and then dividing the amplitude of the second pulse by the amplitude of the first. Accepted resting membrane potentials averaged −68 mV in the soleus and −74 mV in the EDL. Only cells with resting membrane potentials more than −60 mV that did not change by more than 10% were considered in the dataset (Chipman et al., 2010). There was no significant difference in resting membrane potential between genotypes.

### Western blot

Soleus muscles were dissected from the hindlimb of WT and HSA^Cre^BMP4^fl/fl^ mice after 30 d of doxycycline administration (to align with the endpoint of our rotarod experiments). Endplate-rich and endplate-deficient sections of the muscles were dissected and lysed separately in RIPA buffer (Thermo Fisher Scientific) containing complete mini protease inhibitor cocktail tablets (Roche Diagnostics). Total protein content was analyzed using a BCA Protein Assay Kit (Pierce, Thermo Fisher Scientific). Total lysates were then subjected to sodium dodecyl sulfate polyacrylamide gel electrophoresis on a 10% gel and transferred to PVDF membranes. Membranes were subsequently blocked with 5% skim milk in Tris-buffered saline 20 (TBST) for 1 h at room temperature. Blots were first incubated in a primary antibody that selectively recognizes BMP4 (1:1,000, ab39973, Abcam) overnight at 4°C. Membranes were then washed three times in TBST and incubated with the appropriate secondary antibody (HRP-linked anti-mouse, 1:5,000, Cell Signaling Technology) for 1 h at room temperature, analyzed using enhanced chemiluminescence (Thermo Fisher Scientific), and developed in a dark room on Amersham Hyperfilm (GE HealthCare). The blots were subsequently stripped of antibodies using a commercially available buffer (Restore Western Blot Stripping Buffer, Thermo Fisher Scientific) for 15 min, blocked with 5% skim milk and then incubated with an antibody against glyceraldehyde 3-phosphate dehydrogenase overnight at 4°C (GAPDH; 1:50,000, ab37168, Abcam). The blots were washed in TBST and incubated with the appropriate secondary antibody (HRP-conjugated anti-rabbit IgG, 1:10,000, Cell Signaling). The blots were then analyzed and imaged with a ChemiDoc imaging system (Bio-Rad Laboratories). Both blots were scanned in ImageJ software, and the level of BMP4 was quantified by dividing the area of the BMP4 bands by their corresponding GAPDH bands.

### Immunofluorescence and imaging

Muscles were pinned at physiological length in cryostat molds and immersed in a 1:2, 20% sucrose to O.C.T Compound (Tissue-Tek, Sakura Finetek) mixture. Muscles were then flash-frozen in a pool of isopentane cooled in liquid nitrogen and stored at −80°C. Muscles were sectioned (20 µm) using a cryostat (Leica) and mounted onto microscope slides. All sections, except those to be immunostained for S58, were fixed in 4% paraformaldehyde (PFA) for 15 min, rinsed three times for 10 min in phosphate-buffered saline (PBS), and then incubated overnight in PBS containing either 0.3% Triton (PBS-T) or 0.01% Triton (in the case of sections stained for SC-71), 10% goat serum, and a select number of primary antibodies including rabbit anti-DsRed (1:1,000, Clontech Laboratories), rabbit anti-BMP4 (1:250, Abcam), mouse anti-SC-71 (1:5, Developmental Studies Hybridoma Bank), and mouse anti S58 (1:5, Developmental Studies Hybridoma Bank). Sections immunolabeled with S58 were incubated without prefixation but were later fixed prior to incubation in secondary antibodies, as above. All sections were then washed in PBS-T and incubated with the appropriate secondary antibodies at 1:500, including Cy3 goat anti-rabbit (The Jackson Laboratories), Cy2 goat anti-mouse (Jackson Laboratories), Cy3 goat anti-mouse (Jackson Laboratory), and DyLight 594 goat anti-mouse IgA (Abcam) in either 0.3% or 0.01% PBS-T, depending on the primary antibodies used. Sections were also labeled with rhodamine-conjugated α-bungarotoxin (BTX; 1:500, Invitrogen) or Alexa Fluor 647-conjugated α-bungarotoxin (1:250, Invitrogen) for 2 h. Slides were rinsed in PBS and mounted in a mixture of 50% glycerol in PBS containing 0.03 mg/ml phenylenediamine to prevent fading and stored at −20°C.

NMJ morphology and innervation were characterized using muscle whole-mount preparations (Harrison and Rafuse, 2020). Briefly, muscles were pinned at physiological length in cryostat molds on cork and fixed in 4% PFA for 15 min. Muscles were then rinsed in PBS, permeabilized in cold methanol for 6 min, rinsed, and submerged in PBS in a Sylgard-coated dish. The proximal tendons were secured in place with insect pins and distal tendons removed. Bundles of muscle fibers were carefully teased apart using fine forceps under a dissecting microscope (ZEISS). Teased muscle bundles, still attached to the proximal tendon, were then incubated and shaken overnight in 0.3% PBS-T containing 10% goat serum and goat anti-mouse synaptic vesicle 2 (SV2) antibody (1:50; Developmental Studies Hybridoma Bank). Sections were then rinsed and incubated in 0.3% PBS-T containing 10% goat serum and goat anti-mouse Cy2 (1:500) and BTX (1:500) for 1 h. Teased muscles were transferred to microscope slides, the remaining tendon removed, and the bundles arranged and then coverslipped in 50% glycerol in PBS containing 0.03 mg/ml phenylenediamine.

To acquire muscle spindle images, the mice were first perfused with 4% PFA. The hindlimbs were dissected, postfixed for 30 min in 4% PFA, and washed three times for 10 min in PBS. Muscles were cryoprotected by placing them in 30% sucrose/PBS at 4°C until they sank, after which they were transferred to cryostat molds and flash frozen, as above. Muscles were sectioned (20 µm) longitudinally using a cryostat (Leica), mounted on microscope slides, and immunostained overnight at 4°C with antibodies against the neurofilament marker, PGP9.5 (1:500; Abcam), and SV2 to label synaptic vesicles (1:50; Developmental Studies Hybridoma Bank) in PBS containing 0.3% PBS-T and 10% goat serum. Sections were subsequently rinsed in PBS and incubated with their corresponding secondary antibodies and BTX for 1 h. Spindles were identified in the longitudinal sections by the characteristic “banding” pattern formed by the Ia afferents.

Imaging was performed on an upright microscope (Leica) utilizing ZenBlue (ZEISS) software or a laser scanning confocal microscope (ZEISS) utilizing Zen 2009 (ZEISS) software. Confocal microscope images were acquired using Argon 458/488nm, DPSS 561nm, and HeNe 633nm lasers, depending on the fluorophores. Pinhole size was always set to 1 Airy unit. Images were incorporated into figures using either Adobe Photoshop (Adobe Systems) or Imaris (for fragments; Imaris) software.

### BMP4 fluorescence intensity measurements

Cross sections of WT soleus muscle immunostained for BMP4 and labeled with BTX were viewed on a Leica upright microscope. To determine an appropriate, consistent exposure time across sections, 10 sections were visualized, at random, using the autoexposure setting in ZenBlue software. The mean autoexposure time was calculated and then manually applied to all sections used for fluorescence intensity measurements. Fluorescence intensity measurements of BMP4 were taken at muscle ends and regions with BTX^+^ endplates from WT soleus muscle sections (*n* = 12 sections per group). The final fluorescence intensities at these locations were determined following subtraction of the background fluorescence.

### Quantification of endplate size, fragmentation, and innervation

Endplate area measurements (described as the area within a circumferential line drawn around the entire NMJ, with area integrated to capture the spread of BTX^+^ AChR clusters) were performed on longitudinal sections of WT and HSA^Cre^BMP4^fl/fl^ soleus muscle labeled with BTX and imaged using an upright microscope (Leica). The area of 50 BTX^+^ endplates/muscle were quantified by delineating a circumferential line encompassing all individual AChR fragments within a single endplate using the contour function in ZenBlue software (Zeiss). This method determined the spread, not the number, of endplates on the surface of the muscle fiber. The area specifically occupied by BTX^+^ AChR clusters was then calculated using the Threshold function in ImageJ.

Z-stacks through entire endplates in whole-mounted WT and HSA^Cre^BMP4^fl/fl^ muscles were imaged with a confocal microscope (Zen 2009 software, ZEISS). The number of individual fragments making up an endplate were quantified from computerized three-dimensional reconstruction of BTX^+^ fragments (Imaris software, surface function).

Quantification of endplate innervation on extrafusal fibers was determined by focusing through BTX^+^ endplates using an upright fluorescence microscope (Leica). For any given BTX^+^ site, it was determined to be fully innervated or partially innervated based on the degree of overlap in SV2 immunostaining. If SV2 immunostaining was present in the same configuration as the BTX labeling, it was termed fully innervated. Endplates with partial colocalization of SV2 and BTX (approximately ≥10% and ≤80% innervated) were classified as partially innervated. Denervated endplates were those with no SV2 colocalization at a BTX^+^ site. Approximately 150 endplates were counted, per muscle, to determine percentage of innervated, partially innervated, and denervated endplates per muscle.

### Cross-sectional area measurements and fiber number measurements

Whole soleus muscle cross-sectional area measurements were obtained from two mid-belly (largest) sections of each muscle. Cross-sectional areas of soleus type I (S58^+^) and type IIa (SC-71^+^) muscle fiber types were quantified from 50 fibers/muscle at endplate-rich regions in WT and HSA^Cre^BMP4^fl/fl^ mice utilizing ZenBlue software. Muscle fiber number was estimated by dividing the average soleus muscle cross-sectional area with the average cross-sectional areas of the individual muscle fiber types.

### Quantification of muscle spindle innervation

Two methods of muscle spindle innervation were utilized. The first method used confocal images taken through the entire muscle spindle to determine whether all the endplates in the spindle were innervated (>80%), whether a subset of endplates were partially innervated (10–80%), or whether the entire spindle was completely denervated. This determination was based on whether there was clear overlap between SV2^+^ immunostaining relative to BTX labeling throughout the spindle.

The second method was used to determine the degree of innervation at individual endplates on intrafusal fibers. This was achieved by quantifying the overlap between the SV2^+^ and BTX^+^ areas on intrafusal fibers as described previously (Briggs et al., 2019). Maximum intensity projections of the *z*-stacks obtained from the SV2 and BTX channels for each innervated or partially innervated spindle (mathematically, we could not include denervated spindles in this analysis) were generated in Fiji (ImageJ). Using the Threshold function, these projections were made binary such that pixels with positive staining appeared as uniform black on a white background. These binary images were then loaded into Adobe Photoshop and overlaid such that the apposed area of SV2 onto BTX appeared a distinct color than the BTX positive region. This new merged image was then reloaded into Fiji. Using a combination of the Threshold and Measure functions, raw area measures were obtained for the SV2 contact area and the BTX positive area. Percent innervation was determined as the proportion of SV2^+^ area overlapping the BTX^+^ area.

### Behavioral tests

P90 mice were trained on the rotarod (Ugo Basile Biological Research Apparatus Company) for 3 d prior to doxycycline administration (designated as baseline). Three days later (designated “Day 1”), rotarod testing was initiated on a semidaily basis for 30 d. On each day of testing, mice performed three trials at 20 rpm and four trials at 40 rpm with 5 min breaks between trials. Latency to fall, in seconds, was recorded for each trial with a maximum time of 100 s. Grip strength measurements were taken using a Grip Strength test (Bioseb–In Vivo Research Instruments), where mice held onto a horizontal bar with their forelimbs while the tail was gently pulled (parallel to the floor) until they released their grip. Grip force was recorded, in Newtons (N) from five consecutive trials per mouse. Baseline grip strength was assessed prior to doxycycline administration and then weekly after on test days 5, 12, 19, and 26 d. The same researcher performed all grip strength measurements to ensure consistency. Horizontal ladder experiments were performed prior to and after 30 d of doxycycline administration. HSA^Cre^BMP4^fl/fl^ (*n* = 5) and WT (*n* = 6) mice were filmed while performing three passes across a horizontal ladder, and the number of hindlimb missteps (i.e., steps between rungs) were counted during a post hoc analysis of the videos.

### Experimental design and statistical analysis

All experiments began on ∼P90 male and female HSA^Cre^BMP4^fl/fl^ mice with age-matched, Cre-negative, littermates as WT controls. Initial experiments examining the phenotype of this mouse line separated results based on sex, but no statistical differences were observed between sexes and thus the data was pooled. If data met the criteria for normal distribution, two-way ANOVAs with Bonferroni’s post-tests, or unpaired *t* tests, were used. The former was used in cases where groups were examined over time while the latter was used to compare individual groups. In instances where the criteria for normality were not met (as determined by *F* test), the nonparametric Mann–Whitney test was used. Data were analyzed using GraphPad Prism 5 software and were considered statistically significant at *p* < 0.05. Experiments reported here were not preregistered. Ns are provided throughout in the figure legends.

## Results

### BMP4 expression in WT and HSA^Cre^BMP4^fl/fl^ mice

**Figure 1.**
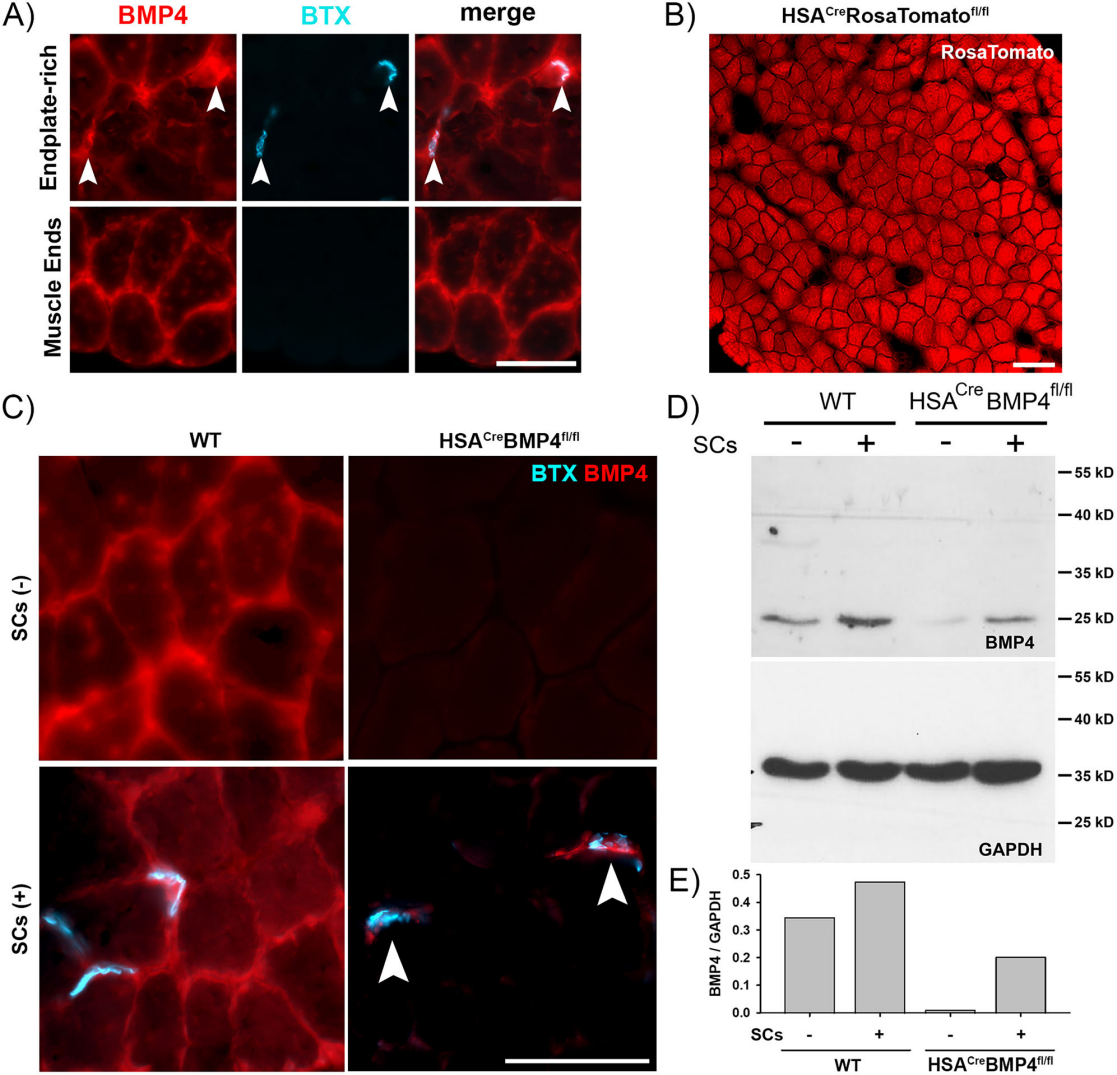
BMP4 expression in WT and HSA^Cre^BMP4^fl/fl^ muscle. ***A***, WT soleus muscle cross sections, immunostained for BMP4 (red) and labeled with BTX (cyan), showing BMP4 expression along the cell membrane and BTX^+^ endplates (arrowheads). Scale bar, 50 µm. ***B***, Soleus muscle cross section from an adult HSA^Cre^RosaTomato^fl/fl^ soleus muscle stained for DsRed (red) shows high tdTomato expression throughout individual skeletal muscle fibers. Scale bar, 100 µm. ***C***, BMP4 immunolabeled (red) and BTX stained (cyan) soleus muscle cross sections from adult WT and HSA^cre^BMP4^fl/fl^ mice showing abundant BMP4 expression in WT, but not in HSA^cre^BMP4^fl/fl^ muscle fibers (top row). BMP4 is retained in SCs at endplates in both genotypes (bottom row; arrowheads). Scale bar, 50 µm. ***D***, Western blots showing BMP4 expression levels in soleus muscle homogenates taken from endplate-rich (+) and endplate-deficient (−) regions in WT and HSA^Cre^BMP4^fl/fl^ mice. GAPDH was used as a loading control. ***E***, The ratio of BMP4/GAPDH expression in WT and HSA^Cre^BMP4^fl/fl^ endplate-rich and endplate-deficient regions indicating that BMP4 muscle fiber expression is lost in HSA^Cre^BMP4^fl/fl^ mice.

Prior to studying the function of BMP4 in skeletal muscle, we first wished to confirm its expression pattern in muscles of adult mice. As reported by Chou et al. (2013), we found that BMP4-immunoreactivity was detected along the sarcolemma of all muscle fibers and at AChR-rich, BTX^+^ endplates in soleus muscle fiber cross sections (Fig. 1*A*). To selectively delete BMP4 expression in adult skeletal muscle fibers, we bred doxycycline-inducible HSA^Cre^ mice with BMP4^fl/fl^ mice to generate HSA^Cre^BMP4^fl/fl^ mice. We chose this cross because Cre recombinase activity is efficiently expressed in skeletal muscle when mice are administered doxycycline, as shown by the RosaTomato expression in the soleus muscle cross section taken from an HSA^Cre^RosaTomato^fl/fl^ mouse administered doxycycline at ∼P90 and killed 2 weeks later (Fig. 1*B*). To confirm that BMP4 expression is eliminated from skeletal muscle fibers after 30 d of doxycycline administration, beginning at P90, we immunostained soleus muscle cross sections from WT and HSA^Cre^BMP4^fl/fl^ mice for BMP4. As shown in Figure 1*C*, BMP4 immunostaining is high in WT, while it is absent in HSA^Cre^BMP4^fl/fl^ muscle fibers. To confirm that BMP4 loss was not restricted to the soleus, we immunostained fast-twitch EDL muscle cross sections from WT and HSA^Cre^BMP4^fl/fl^ mice (Fig. S1). As expected, BMP4 was expressed in WT EDL muscles, while BMP4 was successfully eliminated from the sarcolemma in the HSA^Cre^BMP4^fl/fl^ EDL. As observed in the soleus, BMP4 expression was retained in SCs at the NMJ (Fig. 1*C*, arrowheads).

To validate our immunostaining results, we performed Western blots to assess the presence of BMP4 protein in soleus muscles taken from P120 HSA^Cre^BMP4^fl/fl^ mice and WT control mice that had been fed doxycycline pellets for 30 d. Given that BMP4 is highly expressed in SCs and is transported along nerves (Chou et al., 2013), the soleus muscle was subdivided into endplate enriched regions, containing a high number of SCs and axons (Fig. 1*D*, +), and endplate-deficient regions containing significantly fewer SCs and axons (i.e., muscle sections close to the proximal and distal tendons and away from the NMJs; Fig. 1*D*, −). As expected, we found that the expression of the active, 24 kD form of BMP4 was highly expressed in WT muscles (Neugebauer et al., 2015; Kim et al., 2019; Hoffmann et al., 2020; Gipson et al., 2023), and its expression was higher at endplate-rich regions (Fig. 1*D*, WT +) compared with endplate-deficient regions (Fig. 1*D*, WT −). In contrast, endplate-deficient regions in HSA^Cre^BMP4^fl/fl^ soleus muscles (Fig. 1*D*, HSA^Cre^BMP4^fl/fl^ −) were virtually devoid of BMP4 expression while some expression remained at endplate-rich regions (Fig. 1*D*, HSA^Cre^BMP4^fl/fl^ +). The ratio of BMP4 blot intensity to GAPDH (the loading control) further illustrates the loss of BMP4 expression in HSA^Cre^BMP4^fl/fl^ muscle segments devoid of endplates (Fig. 1*E*). Combined with the absence of BMP4 immunostaining in the HSA^Cre^BMP4^fl/fl^ soleus, the residual expression of BMP4 in endplate-deficient muscle lysates is likely due to the presence of Ib afferents innervating Golgi tendon organs and not from expression of BMP4 in muscle fibers. Taken together, these results reconfirm that BMP4 is expressed in adult skeletal muscles (Chou et al., 2013) and its expression can be eliminated in doxycycline-induced adult HSA^Cre^BMP4^fl/fl^ mice after 30 d of treatment.

### Contractile force of soleus muscles is reduced in HSA^Cre^BMP4^fl/fl^ mice after doxycycline administration

**Figure 2.**
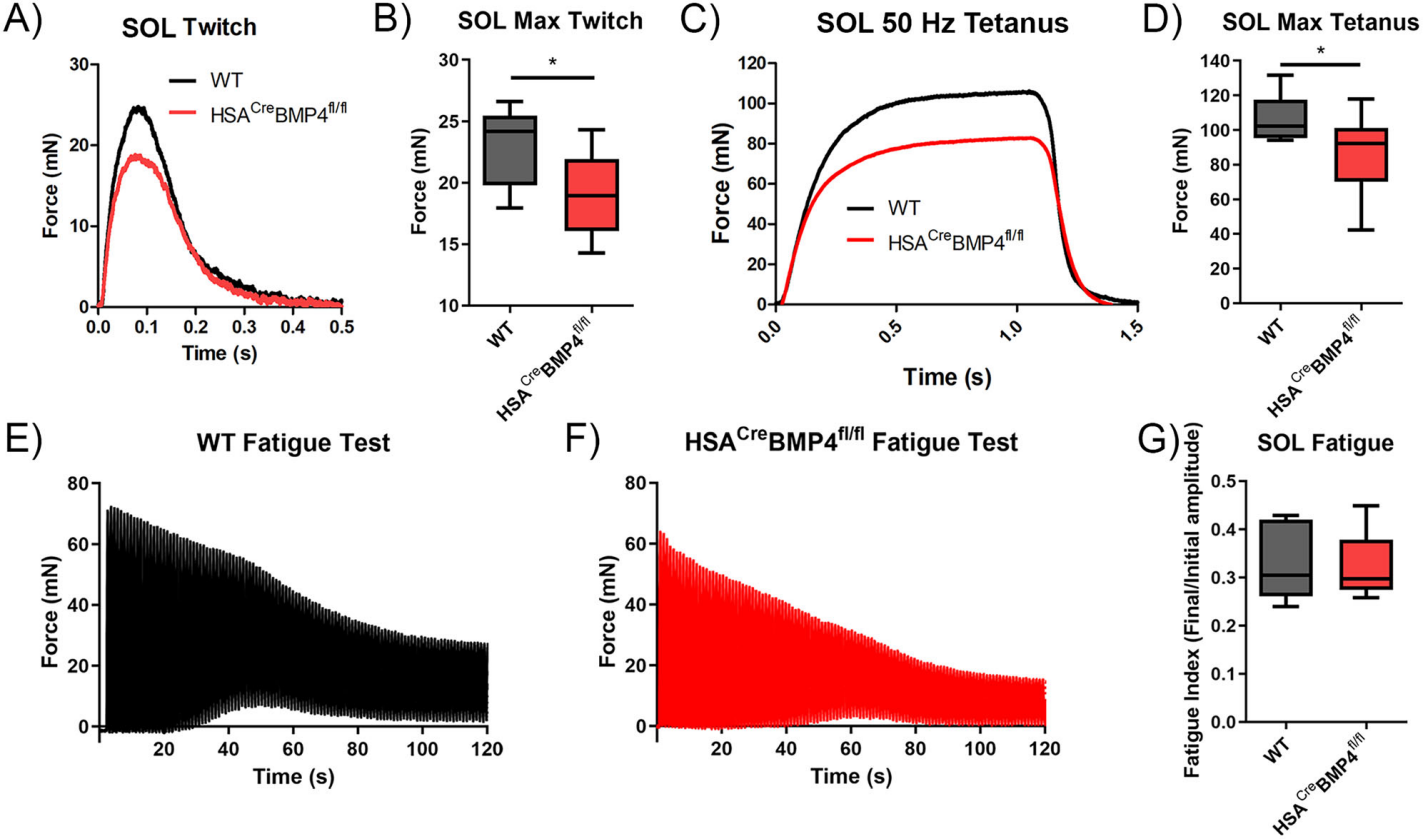
Loss of BMP4 in skeletal muscle fibers leads to a decrease in soleus muscle contractile force. ***A***, ***C***, Representative soleus twitch (***A***) and tetanic (***C***) forces (50 Hz stimuli, 1 s duration) from WT and HSA^Cre^BMP4^fl/fl^ mice, 4 months of doxycycline administration. ***B***, ***D***, Box and whisker plots of soleus twitch (***B***) and tetanic (***D***) forces after 4 months of doxycycline treatment (*p = 0.0465 WT twitch vs HSA^Cre^BMP4^fl/fl^ twitch, *n* = 6; *p = 0.0388 WT tetanus vs HSA^Cre^BMP4^fl/fl^ tetanus, Mann–Whitney test). ***E***, ***F***, Representative WT (***E***) and HSA^Cre^BMP4^fl/fl^ (***F***) soleus fatigue test traces. ***G***, HSA^Cre^BMP4^fl/fl^ and WT soleus (*n* = 6) muscle fatigue indices 4 months after doxycycline administration showing no differences in muscle fatigability (Mann–Whitney test). All box and whisker plot error bars represent min to max.

To determine to what extent the loss of muscular BMP4 in HSA^Cre^BMP4^fl/fl^ mice contributes to skeletal muscle strength, we used ex vivo recordings to measure contractile force of soleus muscles from 210-d-old (P210) HSA^Cre^BMP4^fl/fl^ and WT mice 4 months after doxycycline administration. This time point was selected because it is after motor performance plateaued on the rotarod (Fig. 7*B*,*C*, below). As shown in the representative muscle twitch recordings (Fig. 2*A*), force was significantly reduced by more than 20% in HSA^Cre^BMP4^fl/fl^ soleus muscles compared with WT controls (Fig. 2*A*,*B*; *p* = 0.0164). Tetanic force (50 Hz, 1 s duration) recordings from HSA^Cre^BMP4^fl/fl^ soleus muscles showed a similar decrease in force compared with WT controls after 4 months of doxycycline administration (Fig. 2*C*,*D*; *p* = 0.0371). We also performed ex vivo force recordings on the fast-twitch EDL and found that there were no differences in EDL twitch (Fig. S1) or tetanic (100 Hz, 1 s duration; Fig. S1) forces between genotypes, indicating a potential differential impact of BMP4 loss from different muscle fiber subtypes.

To examine whether fatigability changes in muscles lacking BMP4, we conducted fatigue tests on soleus and EDL muscles from HSA^Cre^BMP4^fl/fl^ and WT mice after 4 months of doxycycline treatment (see Materials and Methods for details). As shown in Figure 2*E–G*, soleus muscles in WT and HSA^Cre^BMP4^fl/fl^ mice had the same fatigue index, indicating that the loss of BMP4 expression did not alter whole muscle endurance during a fatigue test (*p* = 0.4429). Similarly, there was no difference in the fatigue indices between genotypes in the EDL (Fig. S1). Together, these results suggest that the loss of BMP4 in adult muscle fibers leads to a decrease in contractile force in the soleus, but not EDL, and does not alter muscle fatigability.

### Muscle fiber-type-specific atrophy in HSA^Cre^BMP4^fl/fl^ mice

**Figure 3.**
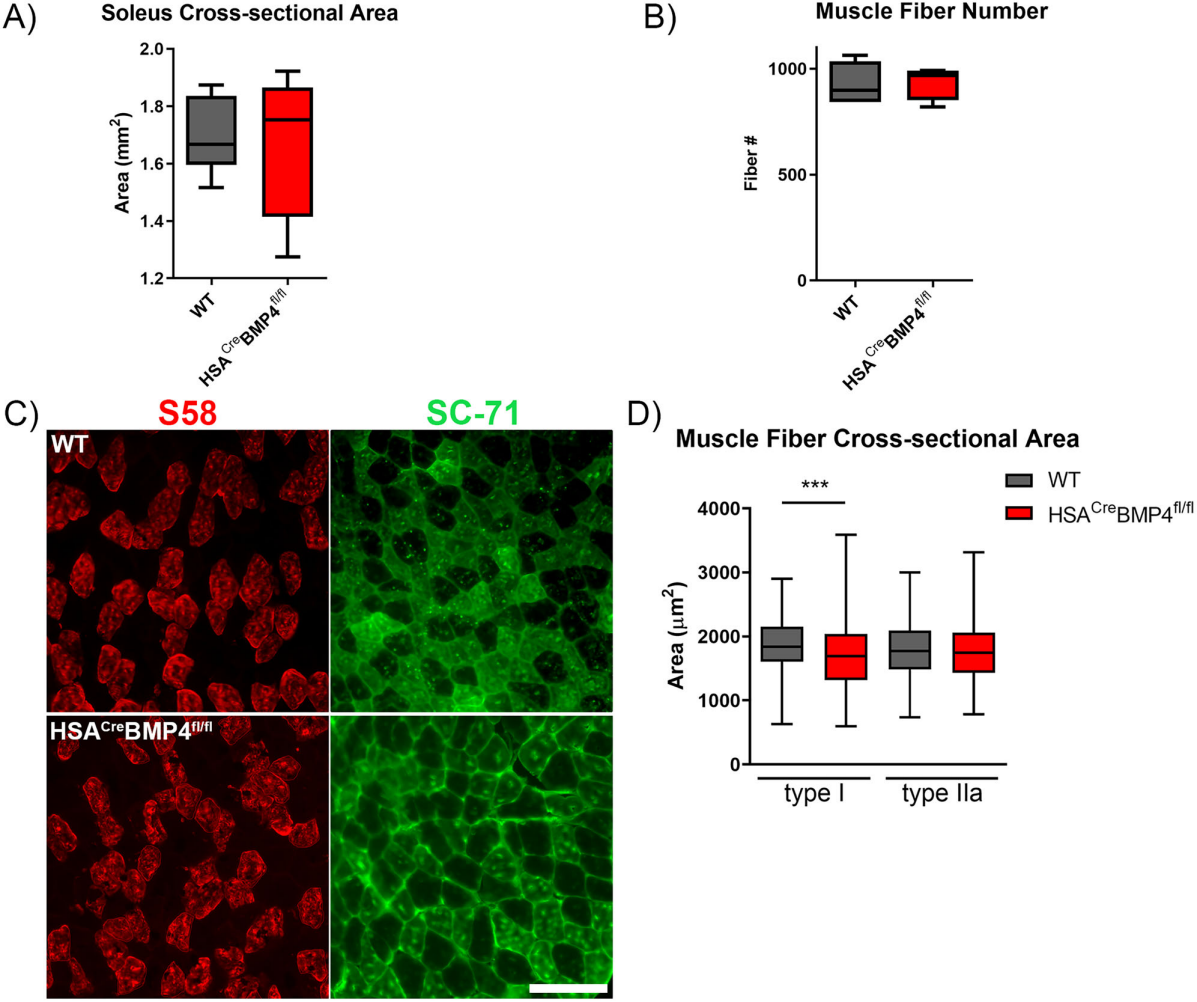
Loss of BMP4 expression in adult skeletal muscle causes atrophy of type I muscle fibers 4 months after doxycycline administration. ***A***, Whole soleus muscle cross-sectional areas (*n* = 8; mm^2^) from WT and HSA^Cre^BMP4^fl/fl^ mice. ***B***, Muscle fiber number at the mid-belly of soleus muscles from *n* = 4 WT and HSA^Cre^BMP4^fl/fl^ mice. ***C***, Representative WT (top row) and HSA^Cre^BMP4^fl/fl^ (bottom row) soleus muscle cross sections immunolabeled for type I (S58; red) and type IIa (SC-71, green) fibers. Scale bar, 100 µm. ***D***, Cross-sectional areas of *n* = 200 type I and type IIa fibers, collected from 4 soleus muscles, per group (i.e., 50 fibers counted per muscle) from WT and HSA^Cre^BMP4^fl/fl^ mice 4 months after doxycycline administration (***p = 0.0003, Mann–Whitney test). All box and whisker plot error bars represent min to max.

Jaime et al. (2024) previously reported decreased myofiber size in soleus type I and IIa fibers following knock-out of the BMP coreceptor, MuSK (Jaime et al., 2024). Therefore, to examine whether changes in muscle fiber cross-sectional area or number occurred in HSA^Cre^BMP4^fl/fl^ mice, we fixed and froze soleus muscles at their physiological length after the conclusion of the tension recording experiments. Muscles were then sectioned for immunohistochemical and anatomical analyses. Figure 3, *A* and *B*, shows that the average whole muscle cross-sectional area and estimated muscle fiber number (calculated by dividing whole muscle cross-sectional area by average muscle fiber cross-sectional area) did not differ between WT and HSA^Cre^BMP4^fl/fl^ soleus muscles, respectively. However, when cross-sectional areas of type I fibers (Fig. 3*C*, S58^+^) and type IIa fibers (Fig. 3*C*, SC-71^+^) were quantified separately, we found that the cross-sectional area of the type I, but not type IIa, fibers were significantly smaller in HSA^Cre^BMP4^fl/fl^ soleus muscles compared with their WT counterparts (Fig. 3*D*; *p* = 0.0003 and *p* = 0.3092, respectively). Taken together, these results indicate that the decrease in contractile force in HSA^Cre^BMP4^fl/fl^ soleus muscles is due, at least in part, to a decrease in type I muscle fiber cross-sectional area.

### The loss of muscular BMP4 causes postsynaptic disorganization in HSA^Cre^BMP4^fl/fl^ mice

**Figure 4.**
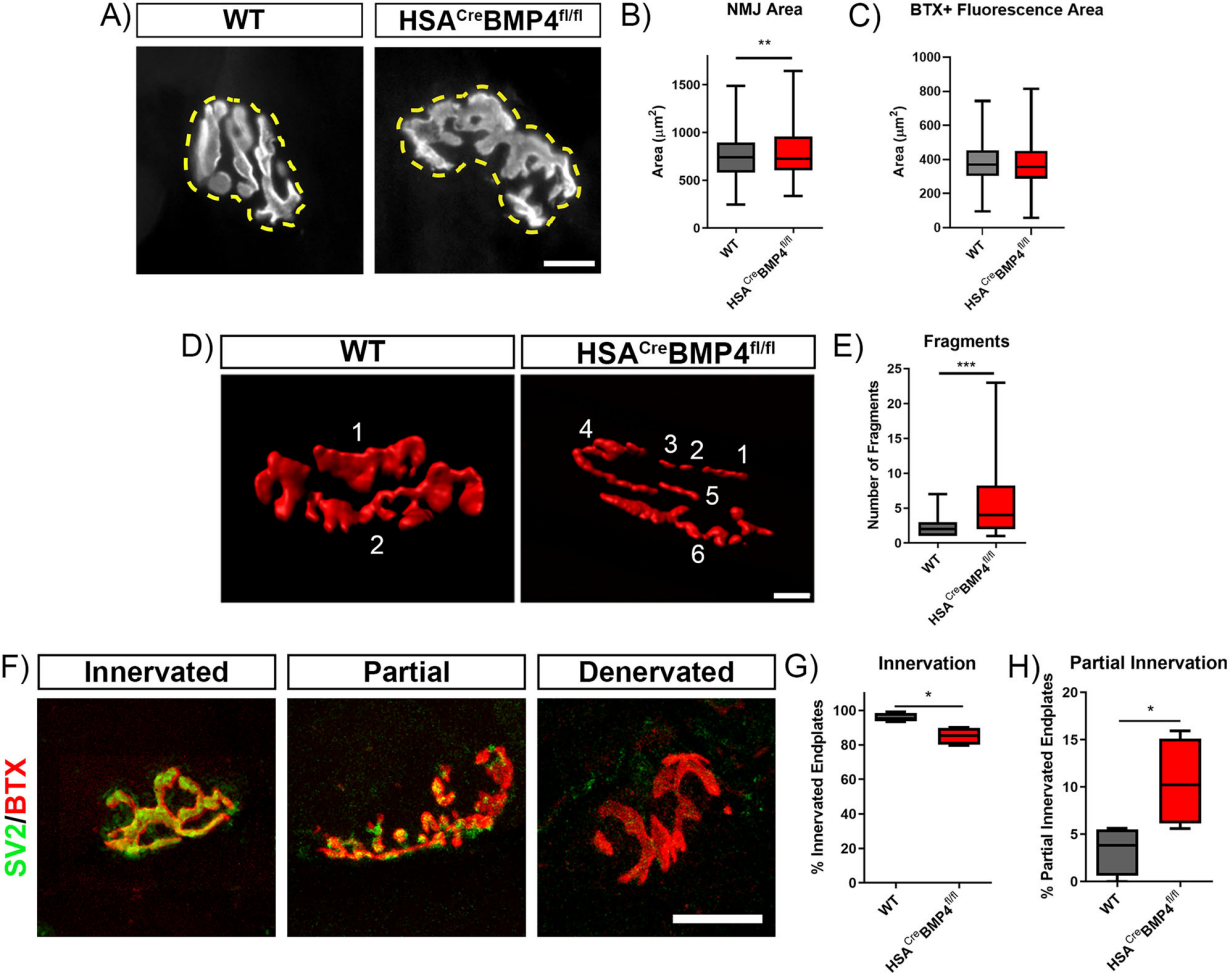
NMJ morphology is altered in HSA^Cre^BMP4^fl/fl^ mice 4 months after doxycycline administration. ***A***, Representative examples of BTX^+^ (white) endplate morphologies in soleus muscles from WT and HSA^Cre^BMP4^fl/fl^ mice. Hashed yellow lines indicate the circumference of all AChRs within a single endplate and how endplate size was measured in figure ***B*** (scale bar, 10 µm). ***B***, Circumferential areas (µm^2^) of WT (*n* = 196) and HSA^Cre^BMP4^fl/fl^ (*n* = 195) endplates observed in four soleus muscles, per group (∼50 endplates measured per muscle; *p = 0.0309, unpaired *t* test). ***C***, BTX^+^ fluorescence area (µm^2^) at WT (*n* = 181) and HSA^Cre^BMP4^fl/fl^ (*n* = 173) endplates from four different mice, per genotype (*p* = 0.9358, unpaired *t* test). ***D***, Examples of volumetric, three-dimensional reconstructions of BTX^+^ (red) endplates rendered from *z*-stacks of whole-mounted WT and HSA^Cre^BMP4^fl/fl^ soleus muscle fibers showing greater fragmentation in the mutant. Numbers indicate the number of fragments (scale bar, 10 µm). ***E***, Number of AChR fragments in individual endplates in WT (*n* = 196) and HSA^Cre^BMP4^fl/fl^ soleus muscles (195; 4 per group, ∼50 endplates per muscle, ***p = 0.0004, Mann–Whitney test). ***F***, Confocal image examples of an innervated (left), partially innervated (middle), and denervated (right) endplate taken from an HSA^Cre^BMP4^fl/fl^ soleus muscle, stained for SV2 (green) and labeled with BTX (red; scale bar, 20 µm). ***G***, Percentage of innervated soleus muscle endplates in WT and HSA^Cre^BMP4^fl/fl^ (*n* = 4) mice per group (*p = 0.0143, Mann–Whitney test). ***H***, Percentage of partially innervated soleus muscle endplates in WT and HSA^Cre^BMP4^fl/fl^ (*n* = 4) mice (*p = 0.0286, Mann–Whitney test). All box and whisker plot error bars represent min to max.

Previous studies in *Drosophila* larvae reported morphological alterations at the NMJ, such as decreased NMJ size and bouton number, when *gbb* was functionally mutated during development (McCabe et al., 2003). To determine whether similar abnormalities occur at vertebrate NMJs when muscular BMP4 is removed in the adult, we quantified NMJ area by labeling HSA^Cre^BMP4^fl/fl^ and WT soleus muscles with BTX after conducting the ex vivo force recordings (i.e., 4 months after doxycycline administration; Fig. 4*A*). The spread of the NMJs (delineated by a circumferential line encompassing all individual AChR fragments; Fig. 4*A*, hashed yellow lines) in HSA^Cre^BMP4^fl/fl^ mice were significantly larger than those in WT mice (Fig. 4*B*). This increase in circumferential area was due to an increase in the spread of the AChR clusters, and not an increase in cluster size, given that AChR fluorescence area (see Materials and Methods for details) was the same between genotypes (Fig. 4*C*). Interestingly, when three-dimensional reconstructions of individual endplates were performed, endplates in HSA^Cre^BMP4^fl/fl^ mice appeared abnormally fragmented (Fig. 4*D*). When quantified, endplates in HSA^Cre^BMP4^fl/fl^ mice were found to be significantly more fragmented compared with their WT littermates (Fig. 4*E*; *p* < 0.0001). These results suggest that the loss of BMP4 expression in adult skeletal muscles leads to a decrease in postsynaptic organization that in turn causes overall endplate area expansion and AChR cluster fragmentation, but not a change in AChR numbers.

To determine whether the highly fragmented endplates in HSA^Cre^BMP4^fl/fl^ mice were accompanied by presynaptic modifications, we immunolabeled and stained teased muscle fibers from both genotypes with SV2 and BTX, respectively. Interestingly, we found examples of fully innervated, partially innervated, and denervated NMJs in both genotypes as illustrated by the representative examples in Figure 4*F*. However, significantly fewer NMJs in HSA^Cre^BMP4^fl/fl^ soleus muscles were innervated 4 months after doxycycline administration compared with their WT counterparts (Fig. 4*G*; *p* = 0.0143). Additionally, significantly more soleus NMJs in HSA^Cre^BMP4^fl/fl^ soleus muscles (*p* = 0.0286) were partially innervated, as defined by the degree of overlap between the pre- and postsynaptic structures (Fig. 4*H*; see Materials and Methods for details). Taken together, these results indicate that BMP4 expression in adult muscle fibers is required for normal organization of pre- and postsynaptic structures.

### Synaptic transmission at the NMJ is altered in HSA^Cre^BMP4^fl/fl^ mice

**Figure 5.**
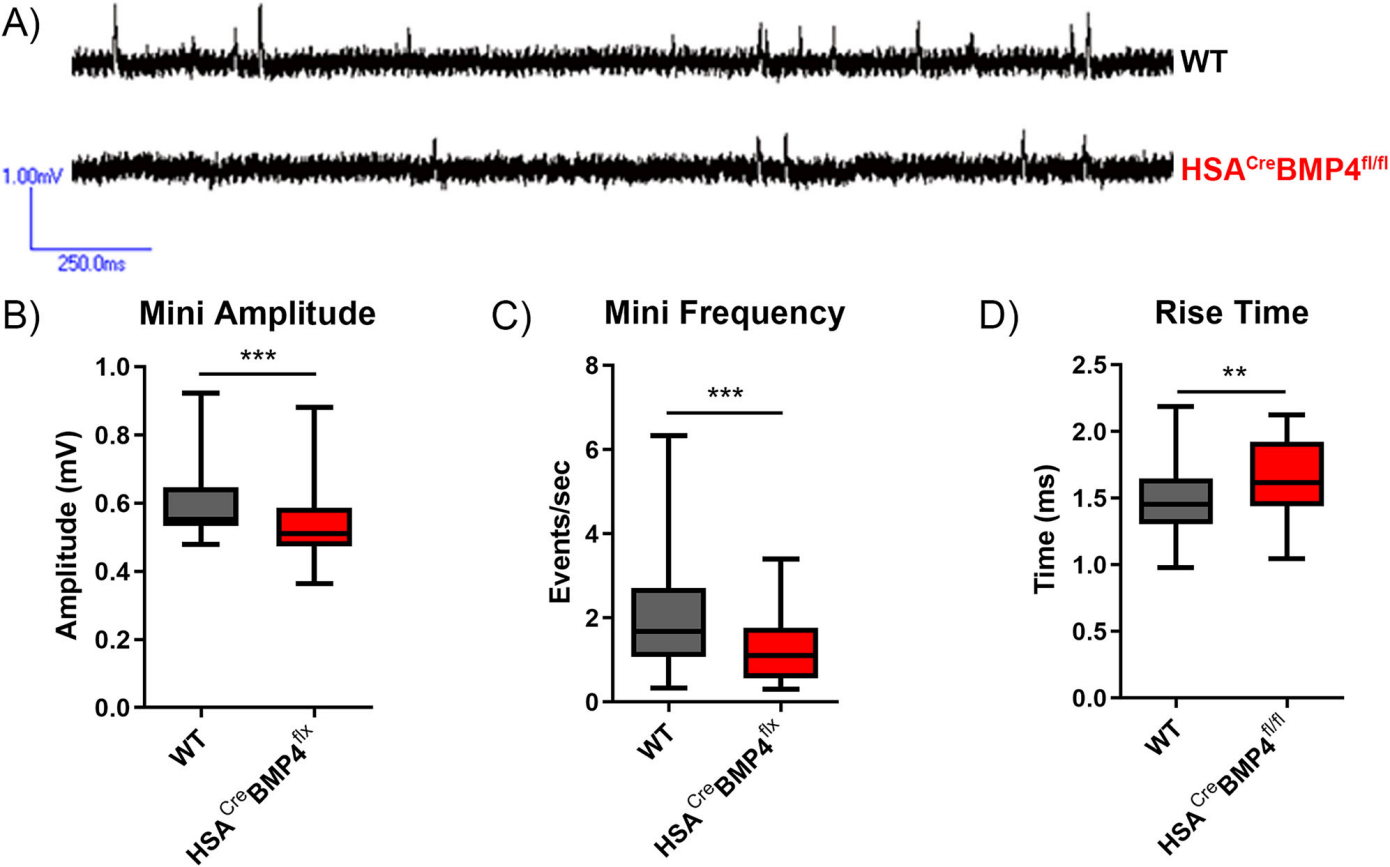
Spontaneous transmitter release is altered in HSA^Cre^BMP4^fl/fl^ soleus muscles 4 months after doxycycline administration. ***A***, Representative traces of mEPPs recorded from soleus NMJs in WT (top) and HSA^Cre^BMP4^fl/fl^ (bottom) mice. ***B–D***, Box and whisker plots of mEPP amplitude (***B***; ***p = 0.004, unpaired *t* test), frequency (***C***; ***p = 0.005, Mann–Whitney test), and rise time (***D***; **p = 0.0025, unpaired *t* test) recorded from soleus NMJs, 4–6 muscles per genotype (*n* = 51 WT and *n* = 47 HSA^Cre^BMP4^fl/fl^ cells included in each dataset). Error bars represent min to max.

Pre- and postsynaptic anatomical defects at the NMJ are often associated with corresponding alterations in synaptic transmission. For example, neurotransmission deficits, indicative of synaptic abnormalities, have been reported in *Drosophila* larvae with mutations in *wit* (Aberle et al., 2002; Marqués et al., 2002) or *gbb* (McCabe et al., 2003). To determine whether similar deficits occur when BMP4 is excised from adult mouse muscle fibers, we conducted sharp electrode electrophysiological recordings of soleus NMJs in P210 WT and HSA^Cre^BMP4^fl/fl^ mice (i.e., 4 months after doxycycline administration). Spontaneous neurotransmitter release was recorded in normal Tyrode’s solution from muscles pretreated with μ-conotoxin to block muscle contraction. The amplitude (Fig. 5*A*,*B*) and frequency of mEPPs (Fig. 5*A*,*C*) were significantly reduced in the mutants (*p* = 0.0004 and 0.0005, respectively). In addition, rise time of mEPPs were also significantly longer in the HSA^Cre^BMP4^fl/fl^ muscles compared with WT (Fig. 5*D*; *p* = 0.0025).

**Figure 6.**
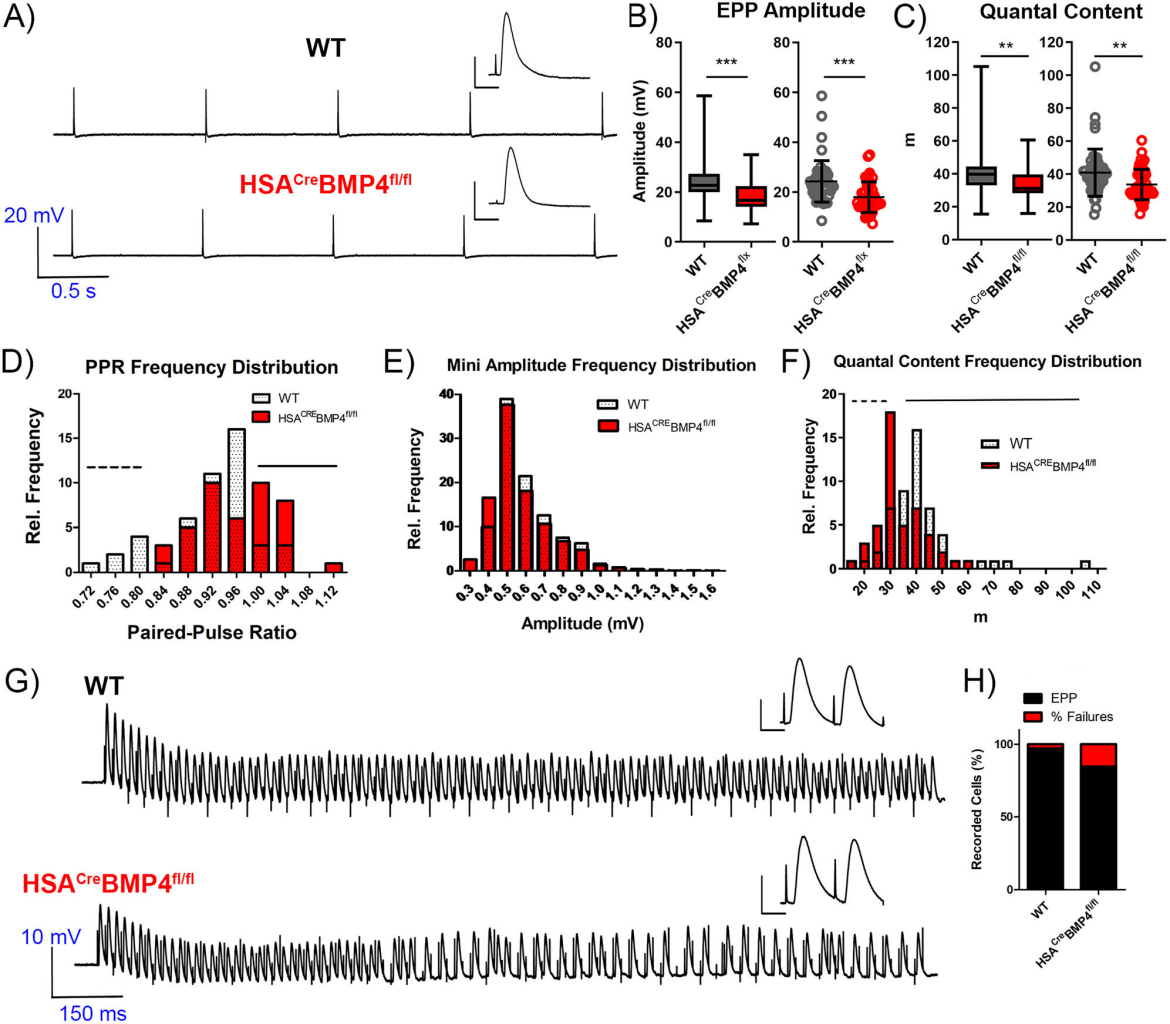
Neurotransmission is altered in HSA^Cre^BMP4^fl/fl^ soleus muscles 4 months after doxycycline treatment. ***A***, Representative traces of evoked EPPs recorded from soleus NMJs in WT and HSA^Cre^BMP4^fl/fl^ mice stimulated at 1 Hz (calibration, 0.5 s, 20 mV). Insets show single EPPs at a shorter time scale (calibration, 10 ms, 10 mV). ***B***, EPP amplitude (represented as box and whisker, min to max, left; and scatter dot, mean ± SD, right, plots) recorded from soleus NMJs from WT and HSA^Cre^BMP4^fl/fl^ mice (***p < 0.0001, Mann–Whitney test). ***C***, Quantal content (m; represented as box and whisker, min to max, left; and scatter dot, mean ± SD, right, plots) for soleus NMJs in WT and HSA^Cre^BMP4^fl/fl^ mice (**p = 0.0011, Mann–Whitney test). ***D***, Paired pulse ratio (PPR) represented as overlaid relative frequency histograms. Hashed line represents absent lower PPR values, and the solid line highlights the gain in higher PPR values in the mutant soleus relative to WT. ***E***, Amplitude of mEPPs plotted as a relative frequency histogram. ***F***, Quantal content from NMJs from WT and HSA^Cre^BMP4^fl/fl^ mice plotted as a relative frequency histogram showing a parallel shift to smaller values in the mutant mice. Hashed line shows the increase in smaller quantal content values while the solid line indicates the decrease in larger quantal contents in the mutant mice, relative to WT. ***G***, Representative traces of evoked EPPs (100 Hz, 1 s duration) recorded from soleus NMJs in WT (top) and HSA^Cre^BMP4^fl/fl^ (bottom) soleus muscles showing multiple failures in the mutant mice. Insets represent examples at a shorter time scale. ***H***, Mean percentage of failures per cell in WT and HSA^Cre^BMP4^fl/fl^ soleus muscles. All recordings taken from *n* = 51 WT and *n* = 47 HSA^Cre^BMP4^fl/fl^ cells, in 4–6 mice per genotype.

Evoked neurotransmitter release was recorded by stimulating the innervating nerve at 1 Hz for 10 s while recording from the NMJ (Fig. 6*A*). Like the mEPPs, EPP amplitudes were significantly reduced in HSA^Cre^BMP4^fl/fl^ soleus muscles compared with WT (Fig. 6*A*,*B*; *p* = 0.0001). Quantal content was also significantly less in the soleus muscles from mutant mice (Fig. 6*C*; *p* = 0.0011). When plotted as a frequency histogram, the overall population of quantal content values recorded from HSA^Cre^BMP4^fl/fl^ soleus endplates (red) shifted to smaller values compared with those recorded from WT littermates (white), indicating that quantal content was reduced in the entire population of NMJs in the mutant mice (Fig. 6*F*, dashed line indicates increase in NMJs with smaller quantal content while the solid line shows a corresponding loss of NMJs with high quantal content). Interestingly, we observed a similar but opposite population shift when we examined paired pulse ratio (PPR). Compared with WT values, the HSA^Cre^BMP4^fl/fl^ PPR showed a shift from smaller (indicated by the hashed line; Fig. 6*D*) to larger values (indicated by the solid line; Fig. 6*A*). There was no difference in the distribution of mEPP amplitudes between WT and HSA^Cre^BMP4^fl/fl^ mice (Fig. 6*E*).

Previous studies on mutant *Drosophila* larvae lacking *wit* also showed a reduction in neurotransmitter release during repetitive stimuli as indicated by an increased rate of failures in Tyrode’s solution containing low extracellular calcium (Marqués et al., 2002). To determine whether neurotransmission is compromised at high rates of activation in our mice, we analyzed the percentage of evoked EPP failures in μ-conotoxin pretreated HSA^Cre^BMP4^fl/fl^ and WT soleus muscles during a 1 s stimulus train at 5, 10, 20, 50, and 100 Hz in normal Tyrode’s solution. While no failures were observed at stimulation frequencies between 5 and 50 Hz, there was a significant increase in the percentage of cells with late-train failures (15.4%) in HSA^Cre^BMP4^fl/fl^ soleus muscle fibers compared with WT (2.86%) stimulated at 100 Hz (Fig. 6*G*,*H*).

Sharp electrode electrophysiological recordings were also performed on EDL fibers from WT and HSA^Cre^BMP4^fl/fl^ mice to confirm that our findings were not restricted to the soleus. The results from the EDL recordings were similar, such that spontaneous transmitter release was compromised in the EDL muscles of HSA^Cre^BMP4^fl/fl^ mice 4 months after doxycycline administration (Fig. S2). As observed in the soleus, mEPP amplitude (Fig. S2) and frequency (Fig. S2) were significantly reduced in the HSA^Cre^BMP4^fl/fl^ EDL compared with WT littermates (*p* = 0.0035 and *p* < 0.0001, respectively). Additionally, rise time of mEPPs were significantly higher in the EDL of mutant mice (Fig. S2; *p* < 0.0001).

EPP amplitudes and quantal content were also significantly reduced in HSA^Cre^BMP4^fl/fl^ EDL muscles relative to WT (Fig. S3). Moreover, while failures in EPPs were not observed in WT EDL muscle fibers stimulated at <50 Hz for 1 s, failures were observed in 7% of the fibers in HSA^Cre^BMP4^fl/fl^ EDL mice when stimulated at 100 Hz (Fig. S3). Interestingly, the frequency distribution of PPR in recorded EDL HSA^Cre^BMP4^fl/fl^ muscle fibers showed a leftward shift to lower values, opposite to the effect observed in the soleus (Fig. S3). This could be due to intrinsic, muscle fiber-type-specific differences in synaptic architecture and functional demands impacting baseline release probability (discussed in detail below).

Taken together, our intracellular recordings indicate that functional neurotransmission at NMJs is compromised when BMP4 is excised from adult skeletal muscle fibers and that these abnormalities occur in both fast- and slow-twitch muscles. Given that the soleus is roughly 40% type I and 60% type IIa (Wigston and English, 1992; Totsuka et al., 2003; Tyagi et al., 2017), and we could not confirm which fiber type we were recording from, these results are not conclusive to indicate muscle fiber-type differences following BMP4 loss. We did not observe two separate populations within our electrophysiological data from the soleus, and similar results were seen in the EDL (primarily composed of IIb fibers), supporting that NMJs from all fiber types were affected by BMP4 loss. These findings indicate that a thorough comparison of the changes in muscle fiber subtypes following BMP4 loss is warranted with future studies.

### HSA^Cre^BMP4^fl/fl^ mice display motor deficits after doxycycline administration

**Figure 7.**
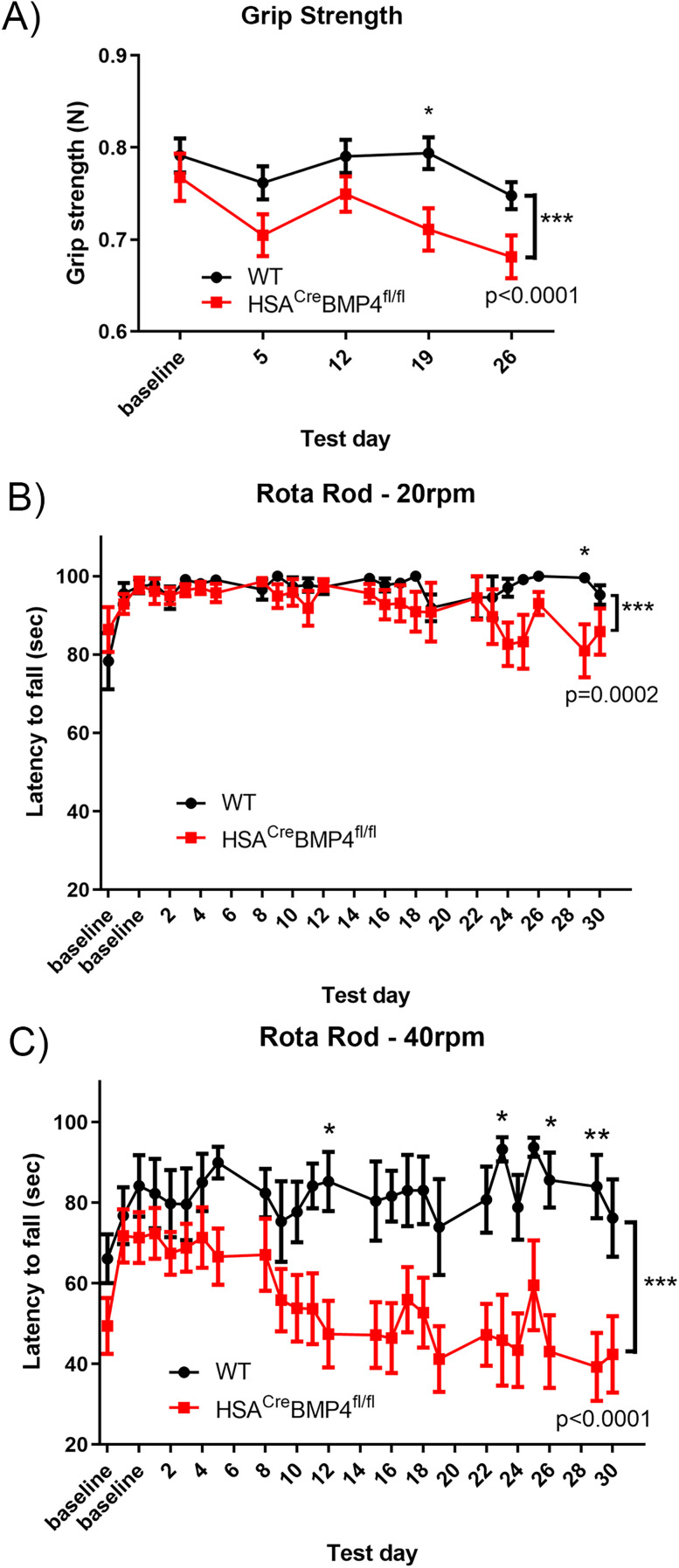
Motor task performance deteriorates over time in HSA^cre^BMP4^fl/fl^ mice. ***A***, Forelimb grip tests from WT (black; *n* = 23–25) and HSA^Cre^BMP4^fl/fl^ (red; *n* = 13–19) mice prior to (baseline) and after doxycycline administration (values shown as mean ± SEM) showing a significant decline in force (N) over time in the HSA^Cre^BMP4^fl/fl^ mice (***p < 0.0001, two-way ANOVA). Post hoc analysis showed an additional individual difference after 19 d of treatment (*p < 0.05, Bonferroni’s post-test). ***B***, Latency to fall off the rota rod at 20 rpm was significantly different between WT (*n* = 13) and HSA^Cre^BMP4^fl/fl^ (*n* = 19) mice (mean ± SEM; ***p = 0.0002, two-way ANOVA) and post hoc analysis showed an individual difference at 28 d (*p < 0.05, Bonferroni’s post-test). ***C***, Rota rod performance at 40 rpm of the HSA^Cre^BMP4^fl/fl^ mice (*n* = 18) declined over time compared with WT (*n* = 10) littermates (***p < 0.0001; two-way ANOVA) and post hoc analysis showed individual differences at 12, 23, and 26–28 d (*p < 0.05, **p < 0.01; Bonferroni’s post-test).

To further characterize the effects of BMP4 excision from adult muscle fibers, we conducted several motor performance analyses. First, we conducted forelimb grip strength tests prior to (baseline), and then weekly after, administration of doxycycline to P90 WT and HSA^Cre^BMP4^fl/fl^ mice. As shown in Figure 7*A*, forelimb grip strength declined over time in HSA^Cre^BMP4^fl/fl^ compared with WT mice (*p* < 0.0001, two-way ANOVA) even though the strength at baseline (i.e., prior to doxycycline treatment) was the same.

To examine whether the loss of BMP4 in adult muscle fibers affects motor control, we conducted rotarod performance tests before and then semidaily after administration of doxycycline for a period of 30 d. We found that HSA^Cre^BMP4^fl/fl^ mice and their WT littermates had similar rotarod performance when the bar was rotated at 20 rpm until Day 23 when the scores significantly deviated (Fig. 7*B*; *p* < 0.002, two-away ANOVA). Performance significantly worsened over time (i.e., decreased latency to fall) when the rotation was increased to 40 rpm and plateaued between 19 and 30 d (Fig. 7*C*; *p* > 0.0001, two-way ANOVA). Post hoc analysis (Bonferroni’s test) showed significant differences at individual time points between genotypes at 12, 23, 26, and 29 d, further confirming the decline in rotarod performance by the HSA^Cre^BMP4^fl/fl^ mice. These results indicate that, in addition to anatomical and electrophysiological abnormalities, the loss of BMP4 expression in adult muscle fibers causes a loss of motor function over time, at least when assessed by grip strength and rotarod performance.

### BMP4 is required for normal muscle spindle structure and proprioception

**Figure 8.**
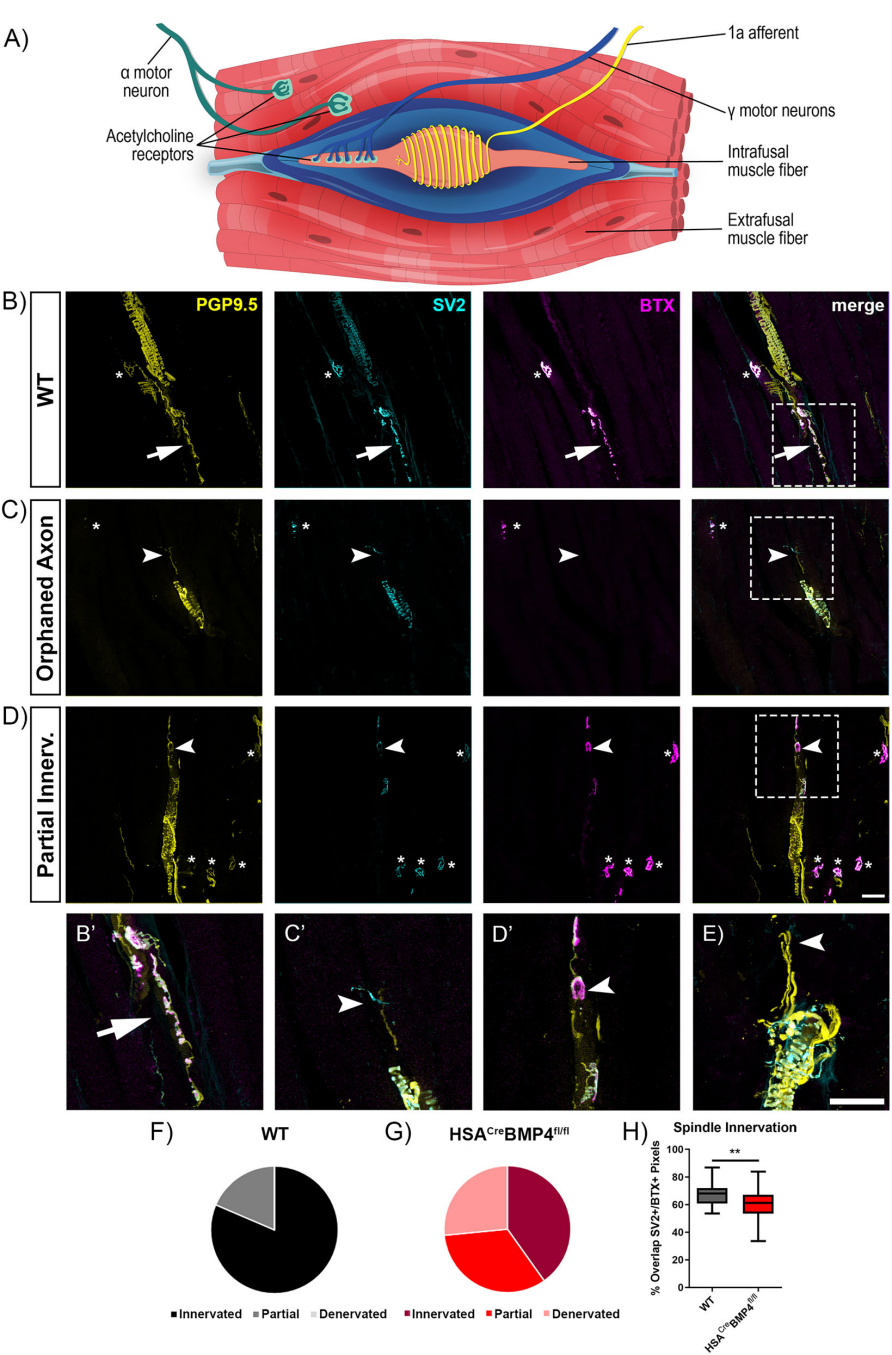
HSA^Cre^BMP4^fl/fl^ mice have morphological abnormalities associated with proprioceptive function. ***A***, Schematic diagram of a muscle spindle showing a single Ia afferent, intrafusal fiber, and alpha and gamma motoneuron. ***B–D***, Longitudinal sections through muscle spindles from WT and HSA^Cre^BMP4^fl/fl^ immunostained for PGP9.5 (yellow) to show the axonal morphologies, as well as SV2 (cyan) and BTX (magenta). Asterisks indicate NMJs on extrafusal muscle fibers. ***B***, Representative confocal images of proprioceptive afferents and gamma motoneurons (arrow) innervating muscle spindles in a WT soleus muscle. ***C***, Example of a HSA^Cre^BMP4^fl/fl^ spindle with absent BTX labeling on the intrafusal fibers and a fragmented gamma motor axon (arrowhead). ***D***, Example of a partially innervated spindle from a HSA^Cre^BMP4^fl/fl^ soleus muscle. ***B’***, ***C’***, ***D’***, Enlarged images of the hashed boxes in ***B***, ***C***, and ***D***, respectively, showing spindle innervation. The overlay of all three colors appears white. Cyan regions (arrowheads) indicate a gamma motor axon without corresponding BTX labeling in ***C’*** and regions of partial innervation in ***D’***. ***E***, Additional example of a HSA^Cre^BMP4^fl/fl^ spindle with no apparent BTX labeling (arrowhead), i.e., denervated. ***F***, ***G***, Pie charts representing the proportion of innervated, partially innervated, and denervated spindles from WT (***F***) and HSA^Cre^BMP4^fl/fl^ soleus muscles (***G***) from four mice (*n* = 35 total spindles examined, per genotype). ***H***, Percent overlap between SV2^+^ and BTX^+^ staining (box and whisker; min to max), converted to pixels, indicating % spindle innervation, obtained at intrafusal endplates from innervated and partially innervated spindles from WT (black; *n* = 27) and HSA^Cre^BMP4^fl/fl^ (red; *n* = 24) soleus muscles (**p = 0.0084, unpaired *t* test). Scale bars, 50 µm.

While significant, we were not convinced that the anatomical and physiological deficits observed in the HSA^Cre^BMP4^fl/fl^ soleus and EDL muscle alone could account for the ∼50% reduction in rotarod performance at 40 rpm. The rotarod performance test is a demanding motor task involving muscle endurance, balance, and proprioceptive feedback (Jones and Roberts, 1968). Skeletal muscles have two proprioceptive receptors important in motor control: muscle spindles and Golgi tendon organs. Muscle spindles consist of Ia afferent sensory neurons and gamma motoneurons innervating multiple intrafusal muscle fibers (Fig. 8*A*). In general, gamma motoneuron activity increases with the complexity of the motor task to fine-tune the spindle’s ability to detect muscle stretch (Prochazka et al., 1988). Given that HSA (the Cre recombinase promoter used in this study) and *Bmp4* are expressed in both extrafusal and intrafusal muscle fibers (Leu et al., 2003; Bornstein et al., 2023), we reasoned that the decline in rotarod performance could be due, in part, to a loss of BMP4 in intrafusal fibers. This loss could lead to reduced proprioception and poorer performance during the rotarod performance test.

To examine this possibility, we first confirmed BMP4 labeling in WT muscle spindles (Fig. S4, left panel, arrowhead) as well as its absence in HSA^Cre^BMP4^fl/fl^ spindles (Fig. S4, right panel, arrowhead), with asterisks indicating extrafusal fibers in both images. We next characterized muscle spindle morphology by staining longitudinal sections of soleus muscles for antibodies against neurofilament, synaptic vesicle protein SV2, and labeled with BTX. As shown in Figure 8*B–D*, the PGP9.5 antibody labels Ia afferents (coiled staining around the intrafusal fiber), alpha-motoneurons (asterisk), and gamma motoneurons (arrowhead). The SV2 antibody labels presynaptic vesicles in both alpha (asterisk) and gamma motoneurons while BTX labels AChRs on extrafusal muscle fibers (asterisk) and intrafusal fibers. Interestingly, we found that some spindles from HSA^Cre^BMP4^fl/fl^ mice had no apparent BTX^+^ AChR labeling, accompanied by wispy or fragmented gamma motor axons, leaving these spindles anatomically and functionally denervated. In addition to completely denervated spindles, we also noticed that some HSA^Cre^BMP4^fl/fl^ spindles contained partially innervated intrafusal muscle fiber endplates (defined as ≤80% overlap between pre- and postsynaptic structures). Two examples of this phenotype appear in Figure 8*C* (arrows, enlarged in 8*C*’) and 8*E* (arrow). When quantified and displayed as pie charts, we found that no WT spindles were completely denervated, while ∼27% of HSA^Cre^BMP4^fl/fl^ spindles had no discernible BTX labeling on intrafusal muscle fibers and therefore no SV2/BTX colabeling (Fig. 8*F*,*G*). In addition to completely denervated muscle spindles, we noticed that spindles in HSA^Cre^BMP4^fl/fl^ mice contained significantly more partially innervated intrafusal endplates compared with their WT littermates (33.3% vs 18.6%; Fig. 8*F*,*G*). When the degree of gamma motoneuron innervation was quantified at individual endplates (by measuring the relative number of SV2^+^ pixels in relation to BTX^+^ pixels; see Materials and Methods for details), intrafusal endplates in the HSA^Cre^BMP4^fl/fl^ soleus muscles were found to be significantly less innervated than WT controls (Fig. 8*H*; *p* = 0.0330).

**Figure 9.**
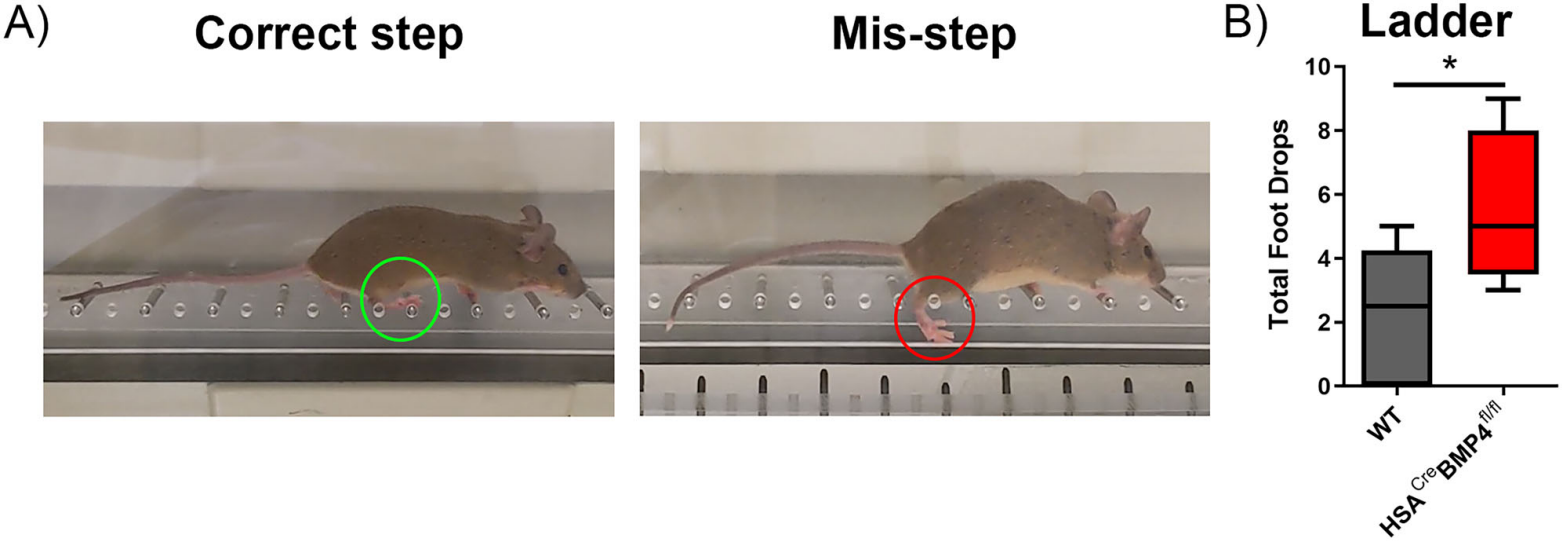
HSA^Cre^BMP4^fl/fl^ mice have behavioral deficits associated with proprioceptive function. ***A***, Example of a HSA^Cre^BMP4^fl/fl^ mouse performing elevated horizontal ladder steps. The left panel shows a correct step where the hindlimb contacts the ladder on a rung (green circle). The right panel shows a foot drop between the rungs (i.e., misstep, red circle). ***B***, Total number of missteps (i.e., steps between rungs) was significantly higher in the HSA^Cre^BMP4^fl/fl^ mice (*n* = 5) compared with their WT (*n* = 6) littermates after completing three passes across a horizontal ladder (*p = 0.0479, Mann–Whitney test). Box and whisker plot error bars represent min to max.

Finally, we performed a horizontal ladder test to further examine whether the gamma motoneuron innervation abnormalities in the HSA^Cre^BMP4^fl/fl^ mice hinders motor control during a task assessing proprioceptive feedback. The horizontal ladder task assesses sensory feedback from muscle spindles and can be quantified by measuring the incidence of rear-foot drops (i.e., stepping between rungs; Fig. 9*A*, right panel) while walking along the ladder (Akay et al., 2014). We first performed baseline ladder experiments on nontreated HSA^Cre^BMP4^fl/fl^ mice compared with WT and found no difference in missteps between groups (data not shown). Thirty days after doxycycline administration, corresponding to when the loss of rotarod performance plateaued, we found that HSA^Cre^BMP4^fl/fl^ mice had a significantly greater number of foot drops compared with their WT littermates (Fig. 9*B*; *p* = 0.0479). Together, these results suggest that BMP4 signaling from intrafusal muscle fibers contributes to normal muscle spindle structure and function and may underlie, in part, the poorer motor performances of HSA^Cre^BMP4^fl/fl^ mice in tasks requiring proprioceptive feedback.

## Discussion

Our results indicate that adult mouse soleus muscles lacking BMP4 are significantly weaker and exhibit fragmented endplates, impaired neuromuscular transmission, and muscle spindle pathologies. Neurotransmission disruptions were observed in both soleus and EDL. These mice also display poor rotarod performance, reduced grip strength, and limb placement on the horizontal ladder. Together, muscular BMP4 contributes to the morphological and electrophysiological stability of vertebrate NMJs as well as muscle spindle innervation in the soleus.

Twitch and contractile forces were ∼20% less in HSA^Cre^BMP4^fl/fl^ soleus, but not EDL muscles, compared with WT controls. This may be due, in part, to reduced cross-sectional area of slow-twitch (type I), but not fast-twitch fibers. These results differ from Sartori et al. (2013) who reported attenuated force in the medial gastrocnemius and decreased size of all oxidative (type I, IIa) and glycolytic (type IIb) fibers following Smad4 deletion. This discrepancy may stem from developmental timing because Smad4 was deleted embryonically in Sartori et al. (2013), while BMP4 was eliminated in the adult, here. Importantly, Smad4 signaling is not only activated by BMP4 but is also a key effector in TGF-β signaling and thus its activity is likely higher in HSA^Cre^BMP4^fl/fl^ mice compared with the ones studied by Sartori et al. (2013). Interestingly, Jaime et al. (2024) reported reduced soleus, but not tibialis anterior myofiber size following deletion of the BMP coreceptor, MuSK, a fiber-type-specific phenotype similar to our preliminary results (Jaime et al., 2024). MuSK was detected in the sarcolemma of slow but not fast fibers, explaining why MuSK loss differentially impacted those fibers (Jaime et al., 2024). BMP4 and AKT-mTOR signaling were also disrupted following MuSK loss (Jaime et al., 2024). Our fiber-type-specific results may similarly be related to the loss of MuSK–BMP4 activity that preferentially impacts slow fibers. These findings further support the role of BMP signaling in maintaining muscle mass (Sartori et al., 2013; Winbanks et al., 2013).

BMP4 loss may impair soleus force disproportionately due to inherent electrophysiological properties. Slow-twitch fibers rely on sustained synaptic input and have limited safety margins, making them more vulnerable to reduced quantal content or vesicle availability (Wood and Slater, 2001; Ermilov et al., 2007). Conversely, EDL type II fibers may compensate via lower baseline release probability, greater synaptic redundancy, or stronger excitation–contraction coupling, even with comparable reductions in EPP and quantal content (Kuno et al., 1971; Molotsky et al., 2022). This suggests BMP4 broadly supports synaptic integrity, but it disproportionately affects muscle fibers requiring sustained, precise neurotransmission.

We observed fragmentation and dispersion of AChR clusters in HSA^Cre^BMP4^fl/fl^ mice, with no change in AChR number, as observed in *wit*-mutant *Drosophila* (Fig. 4*A–E*; Aberle et al., 2002). Why this occurs is currently unknown, but BMP4 presence at the NMJ (Chou et al., 2013) suggests a role in AChR clustering, possibly via the BMP coreceptor MuSK, which regulates AChR distribution (DeChiara et al., 1996; Glass et al., 1996; Lin et al., 2001; Kim et al., 2008; Zhang et al., 2008; Barik et al., 2014; Fish et al., 2025). PDZRN4, which regulates AChR and MuSK localization, also causes AChR fragmentation in knock-out mice (Ham et al., 2025). Therefore, BMP4 may participate in a MuSK–PDZRN4 cascade to maintain AChR clustering.

Our findings of reduced amplitude and frequency of mEPPs, amplitude of EPPs, and quantal content in HSA^Cre^BMP4^fl/fl^ soleus and EDL muscles are consistent with *Drosophila wit* or *gbb* mutants (Aberle et al., 2002; Marqués et al., 2002; McCabe et al., 2003). *Wit* is expressed by motoneurons (Aberle et al., 2002; Marqués et al., 2002) during embryogenesis, and its loss causes a reduction in mEJP frequency, EJP amplitude, quantal content, and increased failure rate (Aberle et al., 2002; Marqués et al., 2002). *Gbb* mutants show similar deficits (McCabe et al., 2003). The milder phonotypes observed here may reflect species differences or the timing of knock-out between models.

Consistent with *Drosophila* studies, we observed increased failures during high-frequency stimulation. While action potential propagation or branch point failures cannot be excluded, previous work has shown complete EPP failures without propagation deficits (Polo-Parada et al., 2001, 2005). The late-train EPP failures observed in HSA^Cre^BMP4^fl/fl^ muscles are more indicative of presynaptic depletion. This may reflect a smaller readily releasable pool, impaired vesicle recycling, or disrupted active zone structure—functions previously linked to BMP signaling (Dobrunz and Stevens, 1997; Zucker and Regehr, 2002; Rizzoli and Betz, 2004). These deficits likely become unmasked under sustained high-frequency stimulation.

Although neurotransmission was disrupted in both soleus and EDL, BMP4 loss produced opposite shifts in PPR in these muscles. This likely reflects differences in synaptic anatomy and functional demands. Synaptic morphology, including terminal size, varicosity of motoneurons, active zone number, and vesicle pool size differ between fast- and slow-twitch muscles and may respond differently to perturbations (Brown et al., 1982; Balice-Gordon and Lichtman, 1994; Somasekhar et al., 1996; Wood and Slater, 2001). Soleus terminals normally release less of their total vesicle pool per impulse, preserving transmission during sustained activity (Reid et al., 1999). The increased PPR we observed suggests reduced initial release probability, leaving a larger readily releasable pool for the second pulse in addition to residual calcium, causing facilitation (Katz and Miledi, 1968; Dobrunz and Stevens, 1997; Zucker and Regehr, 2002). This interpretation aligns with our findings of decreased quantal content and mEPP frequency in HSA^Cre^BMP4^fl/fl^ mice, indicating fewer vesicles are released initially and a role for BMP4 in supporting vesicle release. Conversely, decreased PPR in the EDL following BMP4 loss suggests increased release probability, possibly reflecting dysregulated release machinery. These reciprocal PPR shifts suggest BMP4 has divergent regulatory role in slow- versus fast-twitch motor terminals, perhaps by tuning vesicle release dynamics in a muscle-specific manner, as seen in *Drosophila* (Lee et al., 2016). While a fast–slow muscle comparison was not our aim, this divergence provides insights into BMP4 function that may inform future studies.

HSA^Cre^BMP4^fl/fl^ mice showed progressive rotarod decline, consistent with BMP pathway disruptions in other species. *Drosophila wit* mutants fail to escape their pupal cases, and zebrafish lacking *gdf6a* show reduced endurance and sprint speed (Marqués et al., 2002; Duval et al., 2014). Despite milder deficit in mice, BMP4 clearly supports normal motor function.

Notably, the modest decline in soleus contractile force without fatigue index changes cannot explain the dramatic rotarod deficits. Since HSA, the promoter we used to drive Cre activity, is also expressed in muscle spindle intrafusal fibers (Leu et al., 2003), and the *Bmp4* gene is expressed in mouse intrafusal fibers (Bornstein et al., 2023), we examined spindle morphology. A third of spindles lacked visible endplates, while another third were only partially innervated. These missing endplates may result from fragmentation, as in extrafusal endplates (Fig. 4*D*,*E*). Given their smaller size, fragmented intrafusal endplates may be undetectable using BTX and light microscopy, possibly explaining proprioceptive impairment seen on the horizontal ladder.

Locomotor patterns rely on spinal interneuronal circuits driving patterned activity of flexor and extensor muscles fine-tuned by sensory feedback from spindles and Golgi tendon organs (Rossignol et al., 2006). Disrupting spindle feedback impairs coordinated locomotion including foot placement on the horizontal ladder (Akay et al., 2014). Our results resemble those findings, suggesting that proprioceptive dysfunction contributes to the rotarod deficit in HSA^Cre^BMP4^fl/fl^ mice.

Compensatory mechanisms could explain our milder phenotypes compared with *Drosophila*. During vertebrate embryogenesis, BMP4 forms heterodimers with BMP7 via its prodomain with the BMP4 prodomain required for stable heterodimerization (Neugebauer et al., 2015; Kim et al., 2019). This supports that BMP4 is necessary and sufficient for this heterodimeric activity and that BMP7 would not function similarly without BMP4 (Neugebauer et al., 2015). Further, the presence of BMP4 remaining in SCs warrants consideration. It is unknown if SC-derived BMP4 is released into the synapse or remains within SCs. However, our Western blot findings indicate no compensatory upregulation of BMP4 from SCs (Fig. 1*E*), as total BMP4 in mutant muscle containing SCs (+) matched that of WT muscle without SCs (−).

Finally, reduced muscle force, motor performance, altered neurotransmission, and smaller muscle fibers in HSA^Cre^BMP4^fl/fl^ mice share features with motoneuron diseases. In ALS, for example, NMJ instability leads to impaired neurotransmission, motor axon withdrawal, muscle weakness, and paralysis (Fischer et al., 2004; Gould et al., 2006; Moloney et al., 2014; Arbour et al., 2017; Martineau et al., 2018; Harrison and Rafuse, 2020). Zebrafish with *gdf6a* mutations have shorter lifespans, reduced swim endurance, disrupted NMJ morphology, and a reduction in spinal motoneurons, paralleling ALS pathology (Duval et al., 2014), which are worsened by superoxide dismutase 1 (SOD1) mutations (Rosen et al., 1993; Duval et al., 2014). Furthermore, BMP pathway activation in proprioceptive neurons alleviated motor deficits in a *Drosophila* ALS model (Held et al., 2019). While further research is required, understanding the proteins and signaling pathways required for NMJ stability will undoubtedly shed light onto how this stability is lost in motoneuron diseases.
